# Analysis of Human Colon by Raman Spectroscopy and Imaging-Elucidation of Biochemical Changes in Carcinogenesis

**DOI:** 10.3390/ijms20143398

**Published:** 2019-07-10

**Authors:** Beata Brozek-Pluska, Jacek Musial, Radzislaw Kordek, Halina Abramczyk

**Affiliations:** 1 Laboratory of Laser Molecular Spectroscopy, Institute of Applied Radiation Chemistry, Lodz University of Technology, Wroblewskiego 15, 93-590 Lodz, Poland; 2 Medical University of Lodz, Department of Pathology, Chair of Oncology, Paderewskiego 4, 93-509 Lodz, Poland

**Keywords:** Raman spectroscopy, Raman imaging, colon cancer, biomarkers, spectroscopic diagnostics

## Abstract

Noninvasive Raman imaging of non-fixed and unstained human colon tissues based on vibrational properties of noncancerous and cancerous samples can effectively enable the differentiation between noncancerous and tumor tissues. This work aimed to evaluate the biochemical characteristics of colon cancer and the clinical merits of multivariate Raman image and spectroscopy analysis. Tissue samples were collected during routine surgery. The non-fixed, fresh samples were used to prepare micrometer sections from the tumor mass and the tissue from the safety margins outside of the tumor mass. Adjacent sections were used for typical histological analysis. We have found that the chemical composition identified by Raman spectroscopy of the cancerous and the noncancerous colon samples is sufficiently different to distinguish pathologically changed tissue from noncancerous tissue. We present a detailed analysis of Raman spectra for the human noncancerous and cancerous colon tissue. The multivariate analysis of the intensities of lipids/proteins/carotenoids Raman peaks shows that these classes of compounds can statistically divide analyzed samples into noncancerous and pathological groups, reaffirming that Raman imaging is a powerful technique for the histochemical analysis of human tissues. Raman biomarkers based on ratios for lipids/proteins/carotenoids content were found to be the most useful biomarkers in spectroscopic diagnostics.

## 1. Introduction

Colon cancer ranks second with regard to the incidence of malignant tumors in women and men and is the third most common cause of death around the world. The mortality rate for this type of cancer is around 60% in the United States and Europe [1]. Etiology and risk factors in colon cancer can be divided into three groups: environmental (a high-fat diet, a high-calorie diet, a diet low in roughage, vegetables and fruits), internal (adenomas, ulcerations, Crohn’s syndrome), and genetic (e.g., familial polyposis) [2]. Every day, the content of the human colon can be described as a diverse mix of bile, mucus, various microorganisms, feed fermentation products, unabsorbed food and its metabolic products including toxins, mutagens, and dissolved gases. That’s why the colon mucosa is constantly exposed to diet- and bacterial-derived oxidants. Such chronic exposure to adverse conditions may then lead to uncontrolled oxidative stress and DNA damage, which can finally cause colon homeostasis and cancer development. Nearly 95% of colon cancers are glandular cancers; the remaining 5% are squamous, mixed, or non-differentiated types [1].

Recently the basis of screening tests in colon cancers is a FOB test (a Fecal Occult Blood test) in the stool in combination with a sigmoidoscopy examination [3]. However, the high mortality indicates insufficient prophylaxis. The basis for the treatment of diagnosed colon cancers is surgery. Surgery for early-stage colon cancer includes removing of polyps during a colonoscopy, endoscopic mucosal resection or laparoscopic surgery; for invasive colon cancer types, most often a partial colectomy or extensively resection of pathologically altered sections of the digestive tract are recommended [4]. The final examination of removed samples, which confirm cancer development, is histopathological analysis based on specific staining and human interpretation. However, the histopathological examination is based on morphological analysis of the sample, does not provide any information about the biochemistry of carcinogenesis, and more advanced techniques of costly immunostaining and molecular biology are needed to receive complex information regarding observed pathological processes. Spectroscopy methods have emerged in recent years as one of the most important alternatives for traditional human tissue examination and the main tools for biomedical applications, and have made considerable progress in the field of clinical assessment.

Nowadays, optical methods, especially those based on vibrational properties of human tissue samples, have proven that the development of new spectroscopic techniques open tremendous possibilities in the detection of cancer changes even if for the early stage of carcinogenesis and that multivariate image-guided spectroscopic diagnosis can provide the objective biochemical information in real-time with entirely automated protocols of suspicious lesions [5,6,7,8,9,10,11,12,13,14,15,16,17,18,19]. Moreover, spectroscopic methods can obtain a medical relevance in the early endoscopic diagnosis of cancer and can also serve as guidance for precise identification of tissue margins during surgical procedures in real-time. Vibrational spectroscopy, including Raman spectroscopy, is one of the oldest methods used in the analysis of biological molecules and samples [20,21,22]. The position, intensity and width of Raman peaks in vibrational spectra can be easily used to monitor functional groups or particular molecules in different conditions and surrounding mediums, including different biological matrices [23,24,25,26,27,28,29]. In biological systems, such different conditions of the samples may be related to health or disease states, proper or disturbed metabolism, stress, etc. Undoubtedly, huge progress in the use of Raman spectroscopy in biomedical applications has been made, thanks to the imaging technique, which allows 2D and 3D spatial analysis [5,6,7,8]. In recent years many papers have been dedicated to the applications of Raman spectroscopy and imaging in biomedical diagnostics. Researchers have shown that results of vibrational analysis of biological samples can lead to the qualitative and quantitative data in rapid, reproducible and low-cost measurements. Many human organs were analyzed using Raman spectra for example: cervix [9], brain [10,11], lungs [12], arteries [13], breast [14,15,16,17,24], bladder [18], esophagus [19], and colon [30]. Raman spectroscopy and imaging has emerged as a significant breakthrough technique in tissue [9,10,11,12,13,14,15,16,17,18,19,20,21,22,23,24,25,26,27,28,29,30,31] and cell analysis [32], including of living cells [32].

In this manuscript, we will discuss the applications of Raman spectroscopy and imaging in colon cancer diagnostics. The publication is a voice in the discussion about the creation of objective protocols in the real-time diagnosis of cancer changes during routine surgery based on vibrational properties of human cancerous and noncancerous tissues. Raman biomarkers of colon cancer changes are also presented. We would like to also emphasize the advantage of the analysis based on the ratios of the intensity of the biochemical component bands of the human colon tissue in comparison to the analysis based on the intensiveness of single bands.

## 2. Results

In this section we will present the results for patients suffering from colon cancer—tubular carcinoma, cancer stage G2. Before we formulate general conclusions that may be useful in medical diagnostics, we will provide data for one of the analyzed patients, patient P3, to discuss the most important research observations.

**Figure 1:**
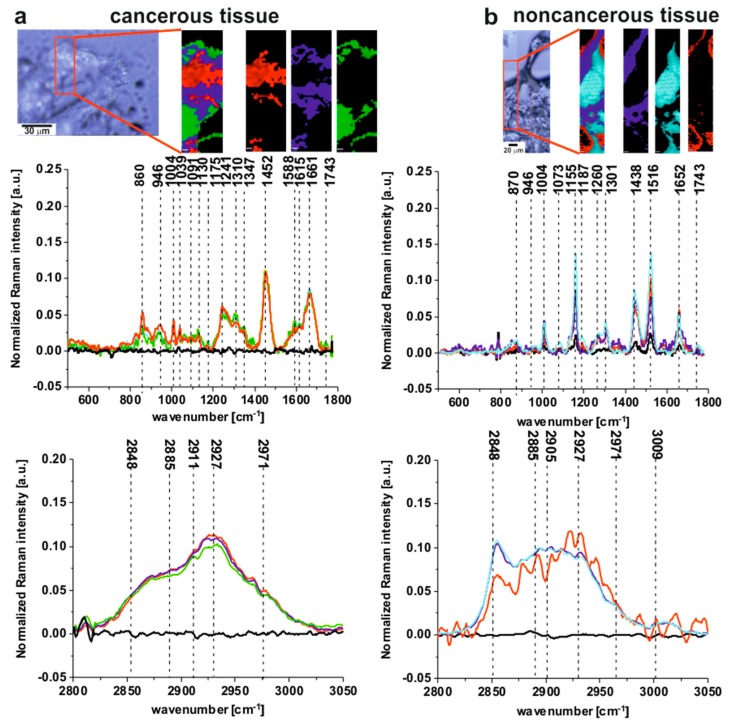
The microscopy image, the Raman image and Raman spectra obtained using Cluster Analysis method for fingerprint and high frequency regions (colors of the spectra correspond to the colors of Raman imaging) for cancerous (**a**) and noncancerous (**b**) tissues of human colon samples (tubular carcinoma, cancer stage G2) from patient P3. Raman image size: 20 × 60 µm and 30 × 150 µm for the cancerous and the noncancerous human colon tissues respectively.

Figure 1 presents the microscopy image, Raman image and Raman spectra obtained using the Cluster Analysis method for fingerprint and high frequency regions (colors of the spectra correspond to the colors of Raman imaging) for cancerous (a) and noncancerous (b) tissues of human colon samples (tubular carcinoma, cancer stage G2) from patient P3.

One can see from Figure 1 that the Raman spectra of the cancerous tissue are dominated by the peaks at 860, 946, 1004, 1039,1091, 1130, 1175, 1241, 1310, 1347, 1452, 1540, 1588, 1615, 1661, 1743, 2848, 2885, 2911, 2927, 2971 cm^−1^, while for the noncancerous tissue peaks at 870, 946, 1004, 1073, 1155, 1187, 1260, 1301, 1438, 1516, 1652, 1743, 2848, 2885, 2905, 2927, 2971, 3009 cm^−1^ are noticed.

All the frequencies mentioned above can be divided into three groups: the nonspecific observed for both types of colon tissues, the typical for the noncancerous tissue and the characteristic for the cancerous type of sample.

Raman bands observed in both the noncancerous and the cancerous tissues can be assigned as follows: 946 cm^−1^—skeletal modes typical for polysaccharides; 1004 cm^−1^—phenylalanine; 1743 cm^−1^—carbonyl feature of lipids and ester groups; 2848 cm^−1^—CH_3_ symmetric stretching modes of lipids; 2885 cm^−1^—CH_2_ antisymmetric stretching modes of lipids and proteins, 2927 cm^−1^—symmetric CH_3_ stretching modes due primarily to protein, 2971 cm^−1^—CH_3_, vibrations of lipids, fatty acids, cholesterol and cholesterol esters [5,6,7,8,9,10,11,12,13,14,15,16,17,18,19,22,23,24,25,26,27,28,29,31,32,33,34].

Table 1 presents the tentative assignments of Raman peaks, which differentiate the noncancerous and the cancerous human colon tissues, including the tendency of concentrations changes of the main chemical components.

To find reliable differentiations between the noncancerous and the cancerous tissues of human colon (data presented for patients P3), we have calculated the average spectra of both types of samples.

**Figure 2:**
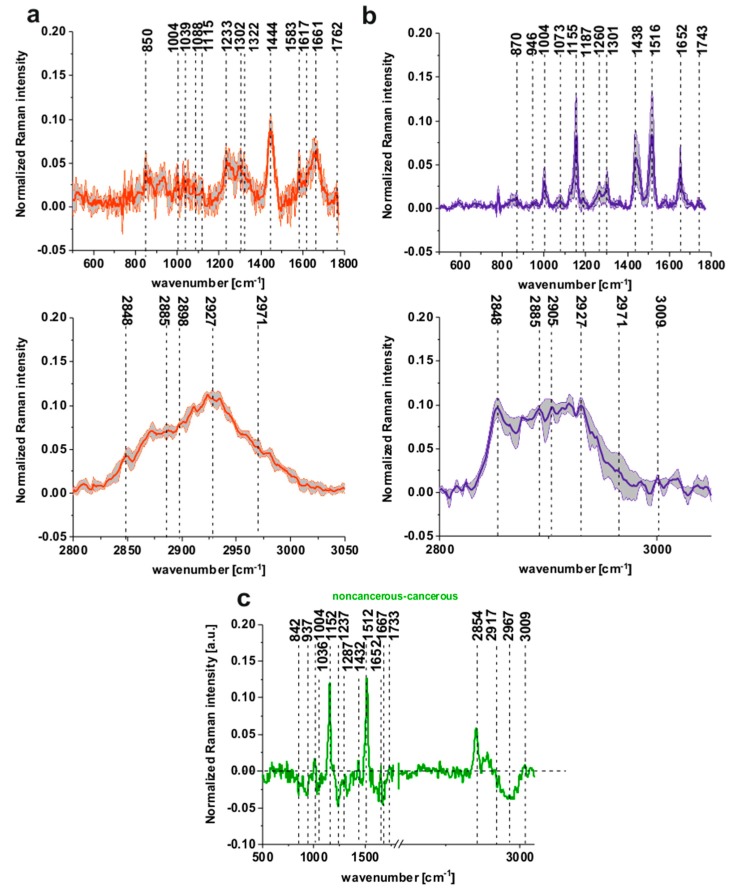
The average Raman spectra based on 20 single spectra of the cancerous (tubular carcinoma, cancer stage G2) tissue (**a**) (red line) and the noncancerous tissue (**b**) (blue line), the differential spectrum between the normalized mean Raman spectra of the noncancerous and the cancerous (tubular carcinoma, cancer stage G2) human colon tissues (**c**), patient P3.

Figure 2a shows the average Raman spectra of tubular carcinoma, cancer stage G2 based on 20 randomly selected single spectra representing all cluster classes seen in Figure 1a of the cancerous tissue, Figure 2b shows the average Raman spectra of noncancerous colon tissue based on 20 randomly selected single spectra representing all cluster classes seen in Figure 1b, patient P3.

To visualize the differences between the noncancerous and the cancerous tissues of the human colon legibly, we have calculated the differential spectrum presented in Figure 2c.

One can see that Figure 2a,b resolves the biomolecular changes associated with cancer transformation, particularly in the spectral ranges of 500–1800 and 2600–3600 cm^−1^.

Figure 2a,b shows that, for the noncancerous tissue, higher contribution in sample composition, compared to the cancerous one, is observed for carotenoids (peaks at 1152, 1512 cm^−1^) [22,23,24,25,26,27,28,29], lipids and fatty acids, including unsaturated fraction (peaks at 1432, 1733, 2854 and 3009 cm^−1^) [33,34,35]. On the contrary, for the cancerous tissue, significant contribution is observed for glucose (peak at 842 cm^−1^) [36], collagen type I (peaks at 937, 1036 cm^−1^) [37], nucleic acids (phosphodiesters group of nucleic acids at 1287 cm^−1^) [38], proteins in beta-sheet conformation (Amide I 1667 cm^−1^ peak typical for structural proteins modes of tumors) [35]; phosphate stretching modes originate from the phosphodiester groups of nucleic acids, which suggests an increase in the nucleic acids in the malignant tissues at 1237 cm^−1^ and methyl groups of lipids and fatty acids, cholesterol and cholesterol esters (peak at 2967 cm^−1^) [35], and nucleosides (790, 852, 1287, 2917 cm^−1^) [39].

To determine statistically significant differences between the noncancerous and the cancerous tissues of tubular carcinoma and to find useful medical diagnosis biomarkers, we have calculated average spectra for both types of samples based on thousands of spectra (36,500).

**Figure 3:**
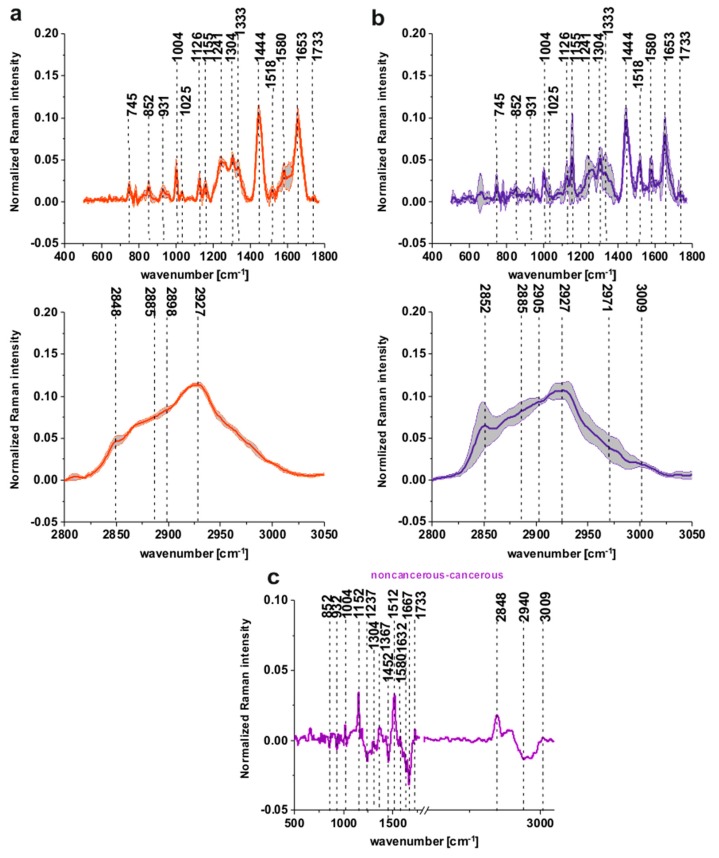
The mean spectra typical for the noncancerous and the cancerous human colon spectra - tubular carcinoma cancer stage G2 based on 36,500 single spectra recorded during Raman measurements of the cancerous (**a**) (red line) and the noncancerous (**b**) (blue line) tissues for patients P1-P5, the differential spectrum between the normalized mean Raman spectra of the noncancerous and the cancerous human colon tissues, tubular carcinoma, cancer stage G2 based on 36,500 Raman single spectra for tubular carcinoma of colon, cancer stage G2 (**c**).

Figure 3a,b shows the normalized mean spectra typical for the noncancerous and the cancerous human colon spectra based on 36,500 spectra recorded during Raman measurements for patients P1-P5 (normalization model: divided by norm).

One can see from Figure 3a,b that the spectra typical for the noncancerous tissues are much more diverse, which confirms the high individual variability of this tissue and that carcinogenesis causes more uniform changes during the development of the pathological process.

Figure 3c presents the differential spectrum between the normalized mean Raman spectra of the noncancerous and the cancerous human colon tissues, tubular carcinoma, cancer stage G2 based on 36,500 Raman single spectra for tubular carcinoma of the colon, cancer stage G2.

The comparison between Figure 2c and Figure 3c shows, once again that for a large group of spectra, once again the main differences between the noncancerous and the cancerous (tubular carcinoma, cancer stage G2) human colon samples are seen for peaks typical of lipids, phospholipids, carotenoids, proteins, phosphorylated proteins, nucleosides, saccharides, DNA. Small differences between Raman peaks positions, as shown in Figure 2c vs. Figure 3c, can be explained by the fact that Figure 3c shows results based on thousands rather than dozens of spectra.

## 3. Discussion

**Figure 4:**
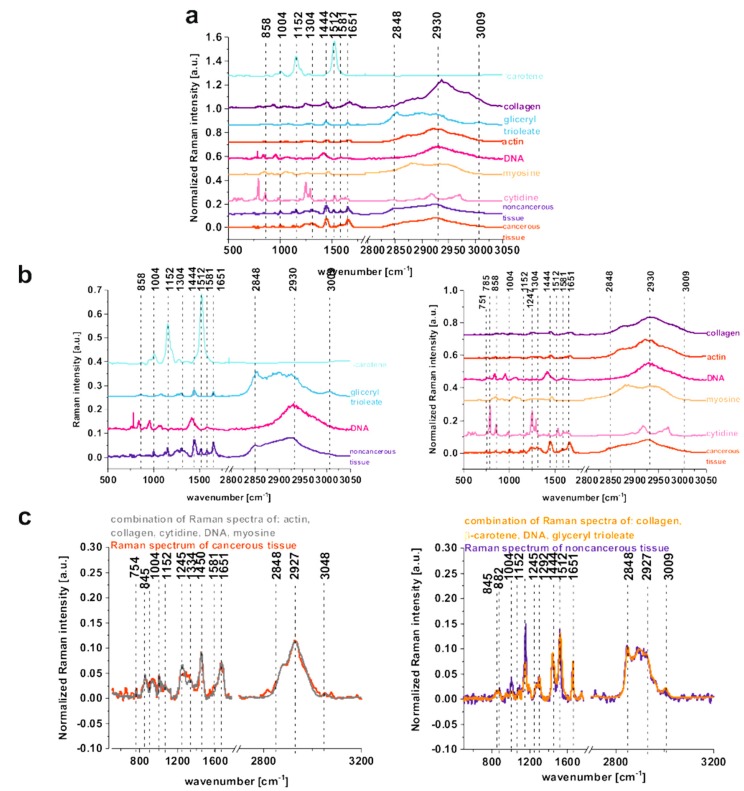
The comparison of the mean Raman spectra of the noncancerous and the cancerous tissues of the human colon (average spectra calculated based on 36,500 single spectra) and Raman spectra of DNA, lipids: glyceryl trioleate, proteins including structural proteins: collagen, actin, myosin, nucleoside molecule: cytidine, and natural antioxidants: beta-carotene (**a**), the comparison of the mean Raman spectra of the noncancerous human colon tissue with beta-carotene, glyceryl trioleate, DNA and the comparison of the mean Raman spectra of the cancerous human colon tissue with collagen, actin, DNA, myosin, cytidine (**b**) the comparison of the Raman spectra of the noncancerous and the cancerous human colon tissue with the combination of Raman spectra calculated based on pure components for the noncancerous and the cancerous human colon tissue (the combination of Raman spectra of pure components was performed using WITec Project Plus) (**c**).

To confirm which individual organic components mainly contribute to the Raman spectra of the tissues, the mean Raman spectra of the noncancerous and cancerous tissues can be compared with typical Raman spectra of: DNA, unsaturated lipids (glyceryl trioleate), proteins including structural proteins (collagen, actin, myosin), nucleoside (cytidine), and natural antioxidants (beta-carotene) in Figure 4a–c.

One can see from Figure 4b that the noncancerous Raman spectrum of the human colon is spectrally similar with the combination of Raman spectra of: beta-carotene, glyceryl trioleate, and DNA, while the cancerous tissue shows similarities with: DNA, collagen, actin, myosin, and cytidine.

DNA contribution, even if taken into account for both types of tissues, is more significant for the cancerous human colon samples for which the high-frequency region is dominated by the peak at around 2930 cm^−1^, and simultaneously the peak at 2848–2852 cm^−1^ is less intense. This observation confirms that abnormal DNA content and hyperchromatic state is typical for the cancerous tissue. When a weak interaction via electrostatic forces between the histones and DNA of the nucleosome components occurs, an alteration of chromatin structure called euchromatin can be observed and positively affects the expression of the oncogenes; the opposite effect can be obtained by the removal of the acetyl groups from the lysine residues, which makes DNA less condensed, resulting in a gene activity repression known as a gene silencing [40,41,42]. The diagnostic role of acetylation and methylation processes by Raman spectroscopy and imaging for human breast cancer has been shown by Abramczyk and coworkers [15]. Table 1 also confirms that peak at 1452 cm^−1^, which is typical for bending modes of methyl groups, is characteristic for malignant tissue and differentiates noncancerous and cancerous samples.

The dominant presence of beta-carotene in the noncancerous human colon tissue shows that carotenoids as natural antioxidants have anti-cancer benefits and can effectively decrease the influence of oxidative stress on colon tissue homeostasis. It has been proved that carotenoids have numerous biological properties and can be considered as chemopreventive agents [22,23,26]. For beta-carotene in colon cancer development, it has been published that incorporating of carotenoid foods into the diet may help to reduce the risk of developing colon and other cancers [22,23,26,43,44,45,46]. The studies of carotenoids with the association to lipids, which are natural reservoirs of these antioxidants, have shown that carotenoids can also play a protective role in lipids oxidation and prevent the intracellular oxidative stress [47]. The significant role of beta-carotene in the prevention of breast cancer and inseparable relationships between carotenoids and lipids including unsaturated fatty acids has been well documented [22,23,24,25,26,27,28,29].

The protective role of oleic acid and his glycerol derivatives in cancer development was also intensively investigated. Numerous studies have reported an inhibition in cell proliferation induced by oleic acid in different tumor cell lines: prostate carcinoma, lung adenocarcinoma and myeloma cell line [48,49]. It has been proved also that oleic acid can induce apoptosis in carcinoma cells [50] and that unsaturated fatty acids via alpha-linoleic or gamma-linolenic acid can promote a positive influence against the carcinogenesis of polyunsaturated fatty acids like eicosapentaenoic acid and docosahexaenoic acid, or can positively stimulate carcinogenesis via arachidonic acid due to inflammations and prostaglandins synthesis [51].

For the cancerous tissue, one can also see an important role played in chemical composition by collagen, actin and myosin. It is well-known that cancer development increases the nuclear-to-cytoplasm ratio in cancerous tissue. The cytological features are commonly used to define the grade of cancer and histological analysis. The most important parameters for such analysis are: the estimation of the degree of anaplasia, the size and shape of cell nuclei, the volume of cytoplasm, the relative number of dividing cells (mitotic index), and the architectural features associated with skeleton proteins. The actin microfilament network is important for cell shape and many cell functions. Adhesion, motility, cellular, signaling cytokinesis and intracellular trafficking of cells depend on actin activity. Myosin belongs to motor active protein, which plays important roles in cytokinesis failure, centrosomal and chromosomal amplification, spindle formation and DNA instability. For carcinogenesis, all the above-mentioned functions of myosin prerequisite tumor formation are important, and subsequently, myosins activate various processes of tumor invasion and metastasis development, including cell migration, loss of cell polarity and suppression of apoptosis [52]. Also, the dominant role of collagen in tumor tissues organization based on human breast cancer is well documented [22,53]. Variation in the cytoskeleton organization, changes in the morphology of cells, motility and adhesiveness are characteristic features of transformed cancer cells. It has been shown in the literature that the cytoskeleton of cells can become promising targets in chemotherapy [54], but detailed analysis of this issue is beyond the scope of this manuscript.

Cytidine, which is a nucleoside molecule formed when cytosine is attached to a ribose ring, plays a special role in colon cancer development and in the biomarkers defining colon cancer. Cytidine has been regarded as a potential biomarker of colon cancer by Hsu W-Y et al. [55]. The Raman spectra of cytidine and cytosine have been reported by M. Mathlouthi and Lee [39,56]. Figure 4b confirms that the Raman spectrum of cytidine explains the vibrational features of cancer mean Raman spectrum especially effective in the fingerprint region. Figure 4c confirms that the combination of Raman spectra of the selected chemical compounds for each type of tissue (noncancerous and cancerous) remains in very high qualitative compliance with the spectra of the human colon.

Because it would be very inconvenient to analyze the whole Raman spectrum during surgery, the intensities of single peaks can vary in a broad range depending on the patient, and because some Raman peaks are observed in spectra of both the noncancerous and the cancerous tissues of human colon, we propose to calculate some ratios for Raman peaks to find parameters that credibly differentiate between the noncancerous and the cancerous tissues.

The ratio of Raman intensities of selected bands, such as 1667/1304, 1452/1667, 1516/1452, and 2848/2940 cm^−1^, are accepted as a good source of information on the ratio of proteins/lipids; proteins (peak typical for malignancy)/Amide I, carotenoids/proteins, and lipids/proteins respectively.

**Figure 5:**
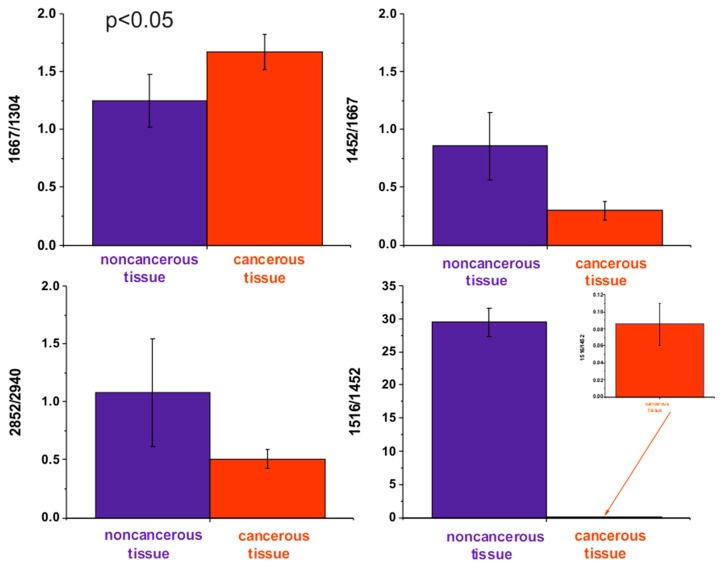
The average ratios of lipids/proteins/carotenoids constituents for the noncancerous and the cancerous human colon tissue samples for tubular carcinoma of the colon, cancer stage G2.

Table 2 and Figure 5 show the average ratios of lipids/proteins/carotenoids constituents for the noncancerous and the cancerous human colon tissue samples for tubular carcinoma of the colon, cancer stage G2, which can be treated as Raman cancer biomarkers.

During the measurements, we were also focused on some technical aspects that could affect the final result and conclusions of our analysis. Because the fresh tissues cut to 16 micrometers can definitely dry relatively fast to check if the Raman spectroscopy of fresh tissue provides reliable results adequate to in vivo studies (where the tissue is hydrated) and to prove that Raman microspectroscopy is a non-destructive technique, we have performed Raman measurements controlling the humidity of sample for the same area of the colon tissue. 

**Figure 6:**
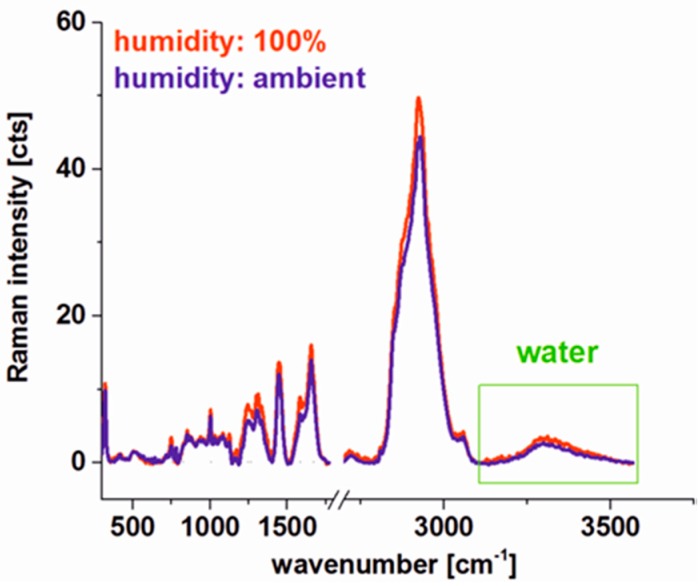
Comparison of Raman spectra for human colon cancerous tissue for 100% and 23% (ambient) humidity conditions.

Figure 6 shows the comparison of average Raman spectra of the human colon cancerous tissue for 100% and 23% (ambient) humidity conditions calculated for the same area of the tissue. For ambient conditions fresh tissue was placed in a cuvette and the Raman imaging was recorded, for 100% humidity the cuvette with tissue was connected to the water reservoir for 24 h. One can see from Figure 6 that the laser beam used during the Raman imaging measurement process does not destroy the tissue. Moreover, Raman peaks intensities of chemical constituents are comparable for 100% humidity (hydrated sample) and ambient humidity conditions—varying less than 10%. For both types of tissues, one can see also broad water peaks in the range 3100–3600 cm^−1^. This observation confirms that for the fresh colon tissue experiments a natural water content, when analysis is performed in a short time after tissue removal, is not distorted and the methodology of human colon Raman microspectroscopy analysis presented in this paper is also a good model for in vivo studies.

## 4. Materials and Methods 

### 4.1. Sample Preparation

Tissue samples were collected during routine surgery. The non-fixed, fresh samples were used to prepare 16-micrometer sections. Specimens of the tissue from the tumor mass and the tissue from the safety margins outside of the tumor mass were prepared for Raman analysis by placing specimens on CaF_2_ windows. Adjacent sections were used for typical histological analysis. All procedures performed in studies involving human participants were in accordance with the ethical standards of the institutional and/or national research committee and with the 1964 Helsinki Declaration. All tissue procedures were conducted under a protocol approved by the institutional Bioethical Committee at the Medical University of Lodz, Poland (RNN/323/17/KE/17/10/2017, 17, October 2017). Written informed consent was obtained from patients. In the manuscript results for 20 samples: 10 cancerous—tubular carcinoma, cancer stage G2 and 10 noncancerous are presented. The selected, single sample was also analyzed using a cuvette to control the humidity. For this sample, the measurement was performed after the cuvette was connected to the tank containing clean water (for obtaining 100% humidity) and in the ambient humidity conditions about 23% after the tank was disconnected. This part of the measurements was performed to demonstrate experimentally that Raman spectroscopy and imaging are non-destructive techniques that enable rapid and objective information on the biochemical composition of analyzed tissues for properly chosen parameters of laser power and integration time. Details of the sample preparation and the research methodology have been described in detail in our previous papers [24,25,26,27].

### 4.2. Raman Spectroscopy and Imaging

All Raman images and spectra reported in this manuscript were recorded using alpha 300 RSA+ confocal microscope (WITec, Ulm, Germany) using a 50 μm core diameter fiber, an Ultra High Throughput Spectrometer and a CCD Camera Andor Newton DU970NUVB- 353 operating in standard mode with 1600 × 200 pixels at −60 °C with full vertical binning. The second harmonic of the Nd:YAG laser—532 nm excitation line was focused on the sample through a 40× dry objective (Nikon, objective type CFI Plan Fluor C ELWD DIC-M, numerical aperture [NA] of 0.60 and a 3.6–2.8 mm working distance). The average laser excitation power was 5 mW, with an integration time of 0.5 s. Rayleigh scattered light was removed using an edge filter. A piezoelectric table was used to record Raman images. Spectra were collected at one acquisition per pixel and 1200 lines per mm diffraction grating. The cosmic rays were removed from each Raman spectrum (model: filter size: 2, dynamic factor: 10), and the smoothing procedure, the Savitzky–Golay method, was also implemented. Data acquisition and processing including baseline removal (model: shape) were performed using WITec Project Plus [24,25,26,27]. All imaging data were analyzed using the Cluster Analysis (CA) method. The use of Cluster Analysis to construct Raman spectral images was pioneered by the re-search group of C. Otto in The Netherlands [28]. Brief Cluster Analysis allows us to group a set of objects (vibrational spectra in our studies) in such a way that objects that are more similar to each other (in the sense of vibrational properties in our case) belong to the same group called a cluster rather than to those in other groups (other clusters). CA was performed using WITec Project Plus, model: Centroid—the k-means algorithm (each cluster was represented by a single mean vector). Some presented data were normalized (normalization model: divided by norm).

### 4.3. Chemical Compounds

Deoxyribonucleic acid from human placenta (D3035), actin from rabbit muscle (A2522), myosin calcium activated from rabbit muscle (M1636), glyceryl trioleate (T7140), cytidine (C4654) were purchased in Sigma-Aldrich and used without any purification in powder form.

## 5. Conclusions

Raman studies of human colon tissue samples for tubular carcinoma, cancer stage G2, show that the vibrational microspectroscopy can provide results that differentiate the noncancerous and the cancerous tissues of the human colon. The biochemical compositions of the noncancerous and the cancerous tissues are different, and the composition of the noncancerous tissue is much more diverse. The content of DNA, cytoskeleton proteins, and lipids, including unsaturated fatty acids fraction, differentiates noncancerous and cancer samples. The multivariate analysis of the intensities of lipids/proteins/carotenoids peaks shows that these classes of compounds can statistically divide the analyzed samples into noncancerous and pathological groups. Ratios of intensities of Raman peaks 1667/1304, 1452/1667, 1516/1452, 2848/2940 cm^−1^ commonly accepted as a good source of information on the ratio of proteins/lipids; proteins (peak typical for malignancy)/Amide I, carotenoids/proteins, lipids/proteins, respectively, can be treated as human colon Raman biomarkers for tubular carcinoma. Raman measurements in a function of humidity has proved that Raman microspectroscopy is a non-destructive technique to analyze the biochemical composition of human colon.

## Figures and Tables

**Table 1:** The tentative assignments of Raman peaks differentiating the noncancerous and the cancerous tissues of human colon samples, including tendency of concentrations changes of main chemical components [5,6,7,8,9,10,11,12,13,14,15,16,17,18,19,22,23,24,25,26,27,28,29,31,32,33,34,35]. Abbreviations: C—cancerous tissue, N—noncancerous tissue of the human colon, ↑—increase in peak intensity.

Wavenumber [cm^−1^]	Tentative Assignments	Type of Human Colon Tissue
860	phosphate group	C ↑
870	most probably the amino acids, polysaccharides collagen	N ↑
1039	collagen	C ↑
1073	triglycerides	N ↑
1091	symmetric PO^2−^ stretching vibration of the DNA	C ↑
1130	palmitic acid, fatty acid	C ↑
1155	C-C (&C-N) stretching of proteins glycogen, carotenoids most likely a cellular pigment	N ↑
1175	cytosine, guanine	C ↑
1187	antisymmetric phosphate vibrations	N ↑
1241	antisymmetric phosphate PO^2−^ (antisymmetric) stretching modes (nucleic acids typical for malignant tissues), the PO^2−^ groups of phospholipids do not contribute to these bands, Amide III (b—sheet and random coils)	C ↑
1260	amide III ν (C-N) and δ (N-H) of proteins	N ↑
1301	CH_2_ deformation of lipids	N ↑
1310	CH_3_/CH_2_ twisting, wagging &/or bending mode of collagens & lipids	C ↑
1347	CH residual vibrations	C ↑
1438	CH_2_ bending mode in normal tissue	N ↑
1452	CH_2_ bending mode in malignant tissues, bending modes of methyl groups (vibrational modes of collagen)	C ↑
1516	beta-carotene C-C stretching mode	N ↑
1540	NH and NH_2_ in cytosine, cytidine	C ↑
1583	phosphorylated amino acids and proteins	C ↑
1615	tyrosine, tryptophan, C=C (protein)	C ↑
1652	amide I ν (C=O) of proteins	N ↑
1661	amide I vibration mode of structural proteins C=C cis, lipids, fatty acids	C ↑
2905	CH stretch of lipids and proteins	N ↑
2911	CH band of lipids and proteins	C ↑
3009	=C-H, lipids, fatty acids	N ↑

**Table 2:** The average ratios of lipid and protein constituents for the noncancerous and the cancerous human colon tissue samples for tubular carcinoma of the colon, cancer stage G2.

Type of Colon Tissue	1667/1304	1452/1667	1516/1452	2848/2940
noncancerous	1.25 ± 0.20	0.86 ± 0.24	29.52 ± 2.14	1.10 ± 0.4
cancerous	1.67 ± 0.13	0.3 ± 0.07	0.1 ± 0.02	0.51 ± 0.07
